# SUPPORT FROM FRIENDS, BUT NOT CHILDREN, BUFFERS AGAINST DEPRESSION AMONG LEFT-BEHIND OLDER ADULTS

**DOI:** 10.1093/geroni/igae098.2892

**Published:** 2024-12-31

**Authors:** Suyi Wang, Minjie Lu, Helene H Fung

**Affiliations:** Beijing Normal University at Zhuhai, Zhuhai, Guangdong, China (People’s Republic); Beijing Normal University at Zhuhai, Zhuhai, Guangdong, China (People’s Republic); The Chinese University of Hong Kong, Hong Kong, China (People’s Republic)

## Abstract

As adult children migrate, old parents are facing the choice of migrating with their children or remaining in their hometowns (being left-behind). Being left-behind is associated with deteriorating psychological well-being among older adults (Adhikari et al., 2011; Song, 2016; Song, 2017; Zhang, 2016). This study aims to examine whether support from children and friends may benefit the well-being of left-behind older adults. The study recruited 52 left-behind older adults (30 females, Mage = 64.96, SD = 5.291), 69 older adults migrated with their children (54 females, Mage = 64.78, SD = 4.465), and 64 older adults who were currently living with their children in the city they grew up (34 females, Mage = 66.28, SD = 5.072, local group). Consistent with previous findings, the results showed that left-behind older adults reported a higher depression level compared to the local group, and the migration group was in-between. In addition, perceived support from children was negatively associated with depression level among the migration group and the local group, but not among the left-behind group. However, only among the left-behind older adults was perceived support from friends living in the same city negatively associated with depression level. The difference in depression level between the left-behind group and the local group was mitigated among participants who perceived strong support from friends. These results highlight the salutary effect of support, especially from friends, on buffering against depression among left-behind older adults.

